# The newfound relationship between extrachromosomal DNAs and excised signal circles

**DOI:** 10.1002/1873-3468.70263

**Published:** 2026-01-03

**Authors:** Dylan Casey, Zeqian Gao, Joan Boyes

**Affiliations:** ^1^ School of Molecular and Cellular Biology, Faculty of Biological Sciences University of Leeds UK

**Keywords:** acute lymphoblastic leukaemia, circular DNA, excised signal circles, extrachromosomal DNA, V(D)J recombination

## Abstract

Elevated levels of extrachromosomal DNAs (ecDNAs) are associated with poor prognoses of many cancer types. These large circular DNAs typically harbour oncogenes and regulatory elements which, together with high levels of ecDNA transcription, confer a growth advantage to cancer cells. Replication of ecDNAs, followed by their unequal distribution at mitosis, further promotes rapid cancer evolution. In contrast to ecDNAs, the role of circular DNA by‐products from V(D)J recombination in cancer development has largely been overlooked. Developing lymphocytes generate millions of excised signal circles (ESCs) each day through gene rearrangement at the immunoglobulin and *T*‐cell receptor loci. Despite their similar size to ecDNAs, ESCs were long assumed to be inert and lost during cell division. However, it is now known that ESCs potently trigger genome instability when complexed with recombinase proteins. Not only this, but new data show that just like ecDNAs, ESCs replicate and persist, with high levels strongly correlating with poor prognosis of *B*‐cell precursor acute lymphoblastic leukaemia (BCP‐ALL). Despite these striking similarities, the properties of ESCs and ecDNAs are seldom linked. Here, we provide the first comparative review of ecDNAs and ESCs, and highlight the reasons why these molecules are more closely related than once assumed.

## Graphical abstract

Extrachromosomal DNAs (ecDNAs) contribute to the progression of many human cancers. In addition, circular DNA by‐products of V(D)J recombination, excised signal circles (ESCs), have roles in cancer progression but have largely been overlooked. In this Review, we explore the roles of ecDNAs and ESCs in cancer development, and highlight why these molecules seem more closely related than once assumed. Created in BioRender. Casey, D. (2025) https://BioRender.com/nojd59p.

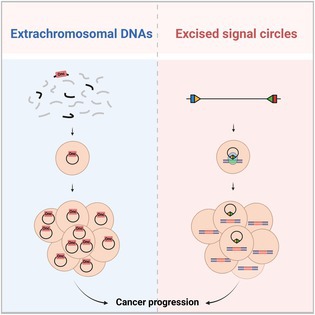

## Abbreviations

**AA**, AmpliconArchitect

**ALL**, acute lymphoblastic leukaemia

**AML**, acute myeloid leukaemia

**aNHEJ**, alternative non‐homologous end joining

**APML**, acute promyelocytic leukaemia

**ATM**, Ataxia‐telangiectasia mutated

**BCP‐ALL**, B‐cell precursor acute lymphoblastic leukaemia.

**BCR**, B‐cell receptor

**BET**, Bromodomain and extraterminal domain

**BFB**, bridge‐fusion‐bridge

**BO**, Barrett's oesophagus

**BrdU**, 5‐bromo‐2′‐deoxyuridine.

**BREC**, B‐cell receptor excision circle

**CfDNA**, cell‐free DNA

**ChIA‐PET**, chromatin interaction analysis with paired‐end‐tag

**CHK**, checkpoint kinase

**CircRNA**, circular RNA

**CJ**, coding joint

**CLL**, chronic lymphocytic leukaemia

**cNHEJ**, classical nonhomologous end joining

**CRISPR**, clustered regularly interspaced short palindromic repeats

**CRISPR‐C**, circularisation of genes and chromosomes by CRISPR

**CRISPRi**, CRISPR interference

**cRSS**, cryptic recombination signal sequence

**CSJ**, chromosomal signal joint

**CSR**, class‐switch recombination

**CtDNA**, circulating tumour DNA

**DDR**, DNA damage response

**DHFR**, dihydrofolate reductase

**DLBCL**, diffuse large B‐cell lymphoma

**DM**, double minute

**DNA**, deoxyribonucleic acid

**DSB**, double‐strand break

**EccDNA**, extrachromosomal circular DNA

**EcDNA**, extrachromosomal DNA

**EGFR**, epidermal growth factor receptor

**EM**, electron microscopy

**ESC**, excised signal circle

**FISH**, fluorescent *in‐situ* hybridisation

**FL**, follicular lymphoma

**HIV**, human immunodeficiency virus

**HJ**, hybrid joint

**HPV**, human papillomavirus

**HPVOPC**, human papillomavirus‐mediated oropharyngeal cancer

**HSR**, homogenously staining region

**Ig**, immunoglobulin

**KREC**, kappa‐deleting recombination excision circle

**LAC**, lung adenocarcinoma

**LAM‐HTGTS**, linear amplification‐mediated high‐throughput genome‐wide translocation sequencing

**LINE**, long interspersed nuclear element

**lncRNA**, long noncoding RNA

**MCL**, mantle cell lymphoma

**MDS**, myelodysplastic syndrome

**MM**, multiple myeloma

**mRNA**, messenger RNA

**NGS**, next‐generation sequencing

**NHEJ**, non‐homologous end joining

**OAC**, oesophageal adenocarcinoma

**PC**, paired complex

**PCC**, postcleavage complex

**PCNA**, proliferating cell nuclear antigen

**PCR**, polymerase chain reaction

**RAG**, recombination‐activating gene

**RNA**, ribonucleic acid

**RNAPII**, RNA polymerase II

**RSS**, recombination signal sequence

**SC**, signal complex

**SCLC**, small cell lung cancer

**sgRNA**, single‐guide RNA

**SJ**, signal joint

**SsDNA**, single‐stranded DNA

**SV**, structural variant

**T‐ALL**, T‐cell acute lymphoblastic leukaemia

**TCR**, T‐cell receptor

**T‐LBL**, T‐cell lymphoblastic lymphoma

**TOP1CC**, topoisomerase 1 cleavage complex

**TOPCC**, topoisomerase cleavage complex

**TREC**, T‐cell receptor excision circle

**WGS**, whole‐genome sequencing

**ψHJ**, pseudo‐hybrid joint

In normal human cells, DNA is neatly packaged into 23 pairs of chromosomes [1]. These structures contain the majority (~99%) of cellular DNA, with the remaining fraction primarily located in mitochondria [2]. In addition, human cells also contain extrachromosomal circular DNAs (eccDNAs) that are distinct from chromosomes and collectively refer to several unique forms which differ in size, function and origin [3, 4].

One form of eccDNA that has been well‐characterised is extrachromosomal DNA (ecDNA)—a large subtype that is extensively characterised in cancer [3]. EcDNAs vary in size from 50 kb to 5 Mb and are found in many types of cancer, where they are linked to increased tumour aggressiveness and reduced patient survival [5, 6, 7]. There are several reasons for this, not least the fact that ecDNAs typically harbour oncogenes. Furthermore, given that DNA circularisation is associated with increased chromatin accessibility [8, 9], transcription of ecDNA‐residing oncogenes is often upregulated compared with their chromosome‐residing counterparts [10]. Taken together, these characteristics imply that ecDNAs are potent molecules, which drive the progression of numerous cancers.

While ecDNA is the predominant cancer‐associated eccDNA, a less well‐characterised form is the by‐product of V(D)J recombination, the excised signal circle (ESC) [11]. During lymphocyte development, V(D)J recombination generates a huge repertoire of immunoglobulin (Ig) and T‐cell receptors (TCRs) from a finite number of gene segments [12]. In doing so, the DNA from in between the gene segments is excised and forms an ESC. While ESCs were initially thought to be inert, it is now known that these molecules potently compromise genome integrity via two related but distinct mechanisms. Studies from Vanura *et al*. [13] and Curry *et al*. [14] first demonstrated that ESCs can trigger genomic damage by reintegrating into the genome through a *trans*‐V(D)J recombination reaction. However, a definitive link with cancer was not confirmed until 16 years later when Balducci *et al*. [15] reported ESC reintegration at genes associated with the development of T‐cell acute lymphoblastic leukaemia (T‐ALL). In complementary studies, Kirkham *et al*. discovered that ESCs, when complexed with the RAG recombinase, cause double‐strand breaks (DSBs) throughout the genome via a cut‐and‐run reaction. Following cutting, the DSBs are released, posing a threat to genome stability and, consistent with this, these breaks were shown to co‐localise with breakpoints associated with B‐cell precursor acute lymphoblastic leukaemia (BCP‐ALL) [16, 17]. However, the true danger of ESCs has only just come to light, with new studies showing for the first time that ESCs replicate and persist through many cell divisions [18]. Heightened ESC replication is linked to increased mutations and BCP‐ALL relapse, demonstrating that, like ecDNAs, ESCs directly cause adverse disease outcomes.

From these discoveries, it now appears that the once considered innocuous ESC plays a defined role in leukaemia progression. This paints a picture whereby, in a similar manner to ecDNAs, ESCs are cancer‐associated eccDNAs. Here, we discuss the history, structure and function of these two circular DNAs before providing an in‐depth analysis of why they appear more closely related than previously believed.

## History, structure and function of ecDNA

Shortly after the discovery of eccDNAs in 1964, Cox *et al*. identified large chromatin bodies in neuroblastoma cell lines [6, 19]. Owing to their unique paired conformations, these molecules were initially given several names: double minutes (DMs), double fragments of chromosome and accessory chromatin [19, 20]. However, it was soon discovered that outside of metaphase, DMs predominantly exist in singlet form [1, 21]. Thus, an alternative term was subsequently adopted: extrachromosomal DNA (ecDNA) [9].

The initial reports from Cox *et al*. [6] gave rise to many questions regarding the molecular characteristics of ecDNAs. Levan and Levan [22] showed that ecDNAs are unresponsive to metaphasic spindle forces, revealing that they do not possess a centromere. Despite their acentric nature, ecDNAs were shown to segregate efficiently into daughter cells by hitchhiking with chromosomes during mitosis [22, 23]. Further experiments in live cells demonstrated that ecDNAs can replicate and that this occurs during S phase independent of chromosomes [24, 25]. Although these foundational discoveries generated extensive insight into ecDNA behaviour, many structural questions remained. While electron microscopy (EM) first indicated that ecDNAs have a nonlinear shape [26, 27, 28], confirmation of circularity was not achieved until much later [23]. To this end, while studying three ecDNA‐harbouring cancer cell lines, the Mischel group utilised a novel bioinformatics tool, AmpliconArchitect (AA), to computationally categorise whole‐genome sequencing (WGS) reads as either linear or circular [8]. Fluorescent *in situ* hybridisation (FISH) confirmed that circular reads were exclusively extrachromosomal. Yet, while this provided strong evidence of nonlinearity, three‐dimensional structured illumination microscopy, combined with transmission and scanning EM, was required to provide unequivocal evidence that ecDNAs truly exist in circular form [8].

In contrast to their structural composition, the link between ecDNAs and cancer was established almost instantly. The initial studies of ecDNAs were largely confined to malignant cell lines, loosely categorising them as cancer‐affiliated entities [23]. In 1978, dihydrofolate reductase (*Dhfr*) gene amplification was shown to mediate methotrexate resistance in murine sarcoma (AT‐3000) and lymphoma (L1210) cell lines [29]. Further investigation in related cell lines revealed a striking correlation between ecDNA levels and increased *Dhfr* copy number [30, 31, 32]. Interestingly, while stable resistance was noted in some cell lines, methotrexate removal reverted others to a methotrexate‐sensitive state [29]. Loss of methotrexate resistance in such cell lines coincided with reductions in ecDNA [30]. However, by continually culturing such cells in the presence of methotrexate, stably resistant phenotypes could be generated [31]. This phenomenon was explained by genomic reintegration of ecDNAs to form homogenously staining regions (HSRs) [33, 34]. HSRs serve as latent ecDNA reservoirs, allowing cancer cells to sequester ecDNAs when selective pressures are removed or decreased [35]. This results in a more stable form of gene amplification, and although allowing increased gene expression, HSRs do not facilitate the same levels of expression as their extrachromosomal counterparts [35]. Subsequent experiments demonstrated that ecDNA‐HSR transition is bidirectional, whereby exposure of stably resistant cells to increasing concentrations of methotrexate regenerated ecDNAs from HSR archives [35, 36].

With an initial link between ecDNAs and cancer established, the race began to further characterise their oncogenic role. As well as drug resistance genes, Von Hoff *et al*. [37] showed that ecDNAs serve as vehicles for oncogenes, demonstrating extrachromosomal *MYC* amplification in cell lines derived from acute promyelocytic leukaemia (APML) (HL‐60) and colorectal adenocarcinoma (COLO320‐DM). Since then, the catalogue of ecDNA‐associated oncogenes has greatly expanded to include *CDK4*, *MDM2*, *ERBB2*, *BRAF*, *KRAS* and *EGFR* (Table 1) [38]. EcDNAs are also now known to harbour regulatory sequences, such as enhancers (e.g. *MYC* enhancer), promoters (e.g. *FGFR2* promoter), long noncoding RNAs (lncRNAs) (e.g. *PVT1*), and transposable elements (e.g. LINE elements) [7, 39, 40, 41, 42, 43]. Immunomodulatory genes (e.g. *LRRC32*) are also frequently identified and allow ecDNA‐harbouring cells to evade immune responses through T‐cell depletion [41, 44]. This implies that ecDNAs promote cancer progression in multiple ways, and while regulatory/immunomodulatory elements often reside within oncogene‐containing ecDNAs, they can also be found within distinct ecDNA species [41, 42]. Moreover, ecDNAs have been detected in many human cancers [5], with a landmark study detecting ecDNAs in more than 80% of the cancer types analysed [45]. Importantly, a strong correlation exists between ecDNA levels and poor patient outcomes, with 5‐year survival rates significantly reduced in patients whose tumours harbour at least one ecDNA [45].

**Table 1:** The reported frequencies of ecDNAs within various cancers and their associated oncogene/drug resistance gene amplifications.

Malignancy	EcDNA frequency (%)	Oncogene/drug resistance gene amplifications	References
Biliary tract cancer	9	*BRAF*, *FGFR2*, *KRAS*, *MAPK1*, *GNB1*	[45, 134]
Bladder cancer	30–50	*CCND1*, *KRAS*, *MDM2*	[45, 135, 136]
Breast cancer	24–40	*CCND1*, *ERBB2*	[45, 137, 138]
Cervical cancer	23	*BIRC3*, *DHFR*, *E6*, *E7*, *ERBB2*, *MYC*	[45, 47, 125, 126, 139, 140]
Colorectal cancer	5	*BRAF*, *DHFR*, *MYC*	[45, 47, 139]
Gastric cancer	27	*FGFR2*, *MYC*	[45, 141]
Glioblastoma	60–76	*EGFR*, *CDK4*, *PDGFRA*	[45, 135]
Haematological malignancies	21	*BRAF*, *BMI1*, *CCND3*, *COMMD3‐BMI1*, *ERBB2*, *KRAS*, *MDM2*, *MYB*, *MYC*, *MYCL*, *NTRK1*, *PRKCI*	[115]
Head and neck cancer	26–39	*ANO1*, *CCND1*, *E6*, *E7*, *EGFR*, *ORAOV1*, *PDL1*, *PVT1*, *SOX2‐OT*, *VOPP1*	[45, 127, 140]
Liver cancer	13	*BRAF*, *KRAS*, *SETDB1*	[45, 142, 143]
Lung cancer	17	*CCND1*, *EGFR*, *MDM2*, *MYC*, *TERT*	[144]
Medulloblastoma	18	*CCND2*, *GLI2*, *MYC*, *MYCL*, *MYCN*, *PPM1D*, *TERT*	[75]
Neuroblastoma	35	*JUN*, *MDM2*, *MYCN*, *SOX11*, *TAL2*	[145, 146]
Oesophageal cancer	38–52	*ERBB2*, *HMGA2*, *MDM2*, *MYC*	[45, 135, 147]
Osteosarcoma	51	*CCNE1*, *CDK4*, *GLI1*	[148]
Ovarian cancer	22–36	*EIF5A2*, *KRAS*, *MDM2*, *PRKCI*, *RHO*, *SKIL*	[45, 149, 150]
Pancreatic cancer	12–29	*BRAF*, *CCND3*, *CDK6*, *KRAS*, *MYC*	[45, 151]
Prostate cancer	2	*AR*	[40, 45]
Rhabdomyosarcoma	33	*AKT3*, *FGFR1*, *MDM2*, *MYCN*, *NCOA1‐PAX3*, *NSD3*, *PAX7‐FOXO1*	[148]
Skin cancer	11–18	*JUNB*, *MYC*, *CALR*	[45, 152]

More recently, there has been a huge shift towards unravelling the additional mechanisms through which ecDNAs promote cancer development. The discovery that ecDNAs harbour enhancer cargo has led to the proposition that they function as mobile enhancers by upregulating expression of chromosomal oncogenes. Key studies also demonstrate a tendency to cluster within cells to form ecDNA hubs. Indeed, such refinements to our understanding of the ecDNA‐cancer landscape have opened the door to new therapeutics, with the pharmacological targeting of ecDNAs currently being explored. These novel insights are further discussed below, but it is clear that despite multiple important discoveries over the last 60 years, the full extent to which ecDNAs promote cancer progression is still being uncovered.

## The formation of ecDNA

**Fig. 1:**
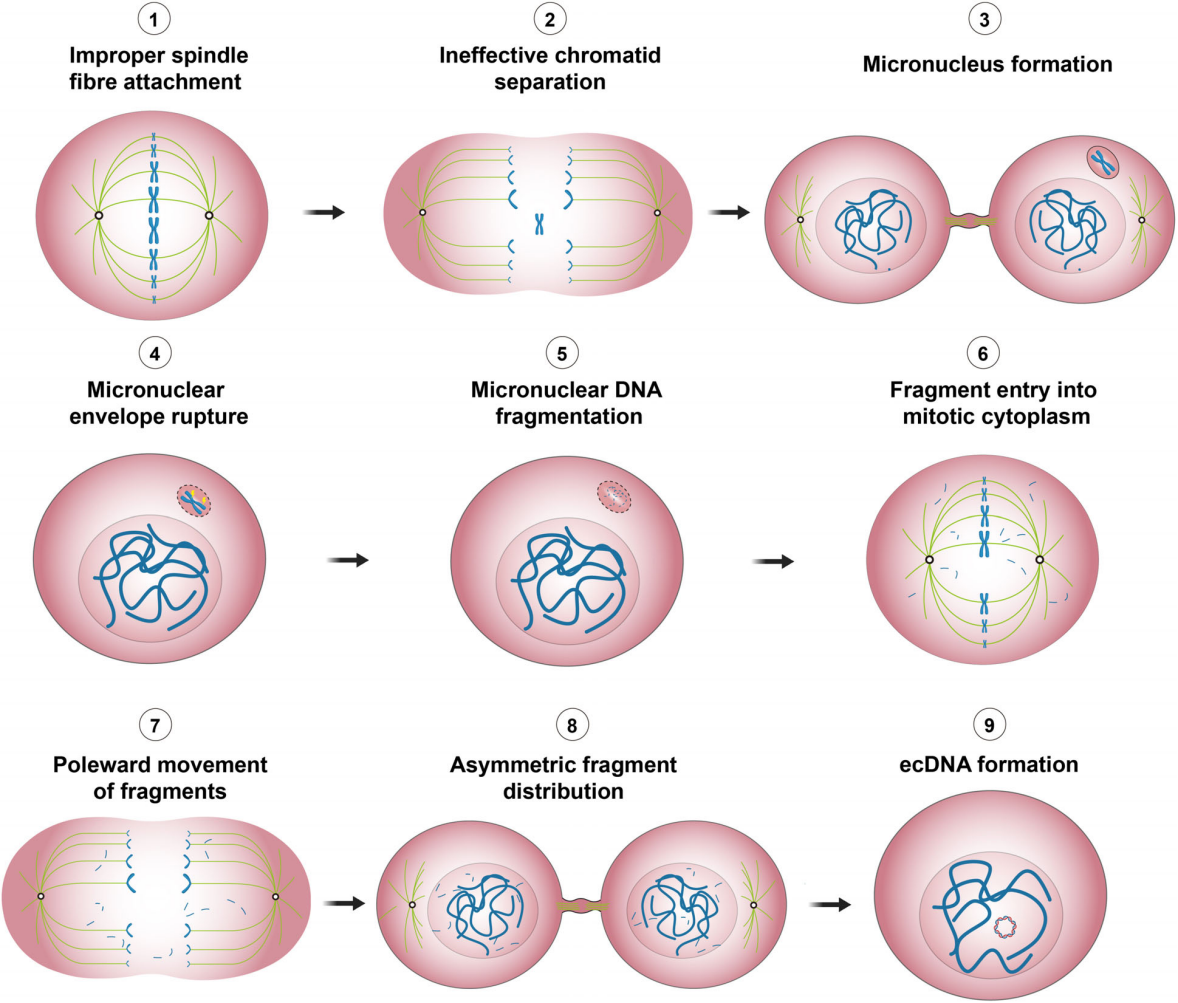
EcDNA formation through chromothripsis. Improper spindle fibre attachment at mitosis perturbs chromatid separation leading to micronucleus formation. The micronucleus is inherited unevenly and is composed of an unstable micronuclear envelope that is prone to collapse. This may expose the sequestered chromosome to cytosolic nucleases, which result in diffuse DSB formation. During subsequent mitosis, the resulting chromosomal fragments reincorporate into the primary nucleus and re‐ligate arbitrarily to generate oncogene‐containing ecDNAs. Figure partially created in BioRender. Wilson, E. (2025) https://BioRender.com/zdonleu.

Since the discovery of ecDNAs, the mechanisms underpinning their generation have been thoroughly investigated but many questions remain unanswered. Several models have been proposed, with the most widely accepted being chromothripsis [46, 47]. Chromothripsis was first implicated in the development of chronic lymphocytic leukaemia (CLL) and results in the shattering of one or more chromosomes [48]. This generates multiple chromosomal fragments which cause gross structural abnormalities within the genome. While inversions and deletions usually predominate, the arbitrary ligation of these fragments may also generate ecDNAs [49]. One chromothripsis model speculates that defective chromosome segregation during mitosis is a causative factor (Fig. 1). During the initial phases of mitosis, improper spindle fibre attachment prevents a chromosome from participating in chromatid separation. While the other chromosomes segregate appropriately, the affected chromosome is inherited by only one daughter cell, becoming sequestered within a micronucleus [50]. Micronuclei are unstable cytoplasmic structures with fragile envelopes that are prone to collapse [51]. Within micronuclei, the precise way in which DNA is fragmented remains elusive. Some evidence indicates that perturbations to the micronuclear envelope expose the chromosome to cytoplasmic nucleases causing diffuse DSB formation [52, 53]. Alternatively, delayed replication timing coupled with premature mitotic entry has been suggested elsewhere [53]. During subsequent mitosis, micronuclear DNA is reincorporated into daughter‐cell nuclei [49, 54], where fragmented chromosomes re‐ligate arbitrarily to form ecDNAs. Indeed, chromothripsis is likely a principal mechanism of ecDNA formation, with one study reporting that 36% of ecDNAs contain signatures characteristic of chromothriptic events [45].

EcDNAs are also proposed to form through breakage‐fusion‐bridge (BFB) cycles that were first discovered in maize by Barbara McClintock [55] and result from the loss of telomeres on chromosomes. During DNA replication, the affected chromosome is duplicated up to the point of telomeric loss. An anaphase bridge then fuses the two sister chromatids together, generating a dicentric chromosome which is pulled apart at mitosis [56, 57]. As the point of breakage does not strictly map to the point of fusion [58], chromosomes inheriting palindromic duplications can arise. Duplications are perpetuated through successive BFB cycles [59, 60], forming chromosomes harbouring HSRs with oncogene amplifications. Chromothripsis and circularisation of these HSRs have been shown to generate ecDNAs [47], and hallmarks of BFB cycling, such as head‐to‐head fold‐back inversions, are present within some ecDNA sequences [5].

The excisional/episomal model proposes that ecDNAs arise through the excision and re‐ligation of chromosomal DNA [9, 34]. This process begins with the generation of an autonomously replicating ‘episome’ which, through subsequent recombination events, gradually enlarges to form ecDNA [34]. As the excised fragment derives from a single chromosomal region, ecDNAs generated through this mechanism are predicted to possess low sequence complexity and little diversity. Further, when chromosomal DNA is excised in the absence of a homologous repair template, the excised fragment giving rise to the ecDNA molecule is perpetually lost from the genome, resulting in a permanent genomic scar not seen for other modes of ecDNA biogenesis [9]. The excisional/episomal model of ecDNA formation is supported by studies of ecDNAs in acute myeloid leukaemia (AML), myelodysplastic syndrome (MDS), and APML [61, 62].

While chromothripsis is widely considered the principal mechanism of ecDNA formation [63], the contribution of others is less clear. Nonetheless, a single unifying factor that appears to predispose cells to ecDNA formation is some form of DNA damage. One school of thought suggests that dysfunction in DNA repair pathways/cell cycle checkpoints is central to this process [21]. Indeed, co‐disruption of the *Brca1* and *Trp53* genes in a mouse breast cancer model resulted in ecDNA formation in 73% of tumours [64]. Similarly, a study investigating the malignant transformation of Barrett's oesophagus (BO) to oesophageal adenocarcinoma (OAC) revealed a strong association between biallelic *TP53* disruption and subsequent ecDNA generation [44]. Taken together, these findings suggest that at least in some cancers, disruption to genomic guardians frequently underpins ecDNA formation [21, 44].

## The intracellular behaviour and fate of ecDNA

Although the formation of ecDNAs is still debated, their intracellular behaviour is relatively well‐characterised. Once generated, ecDNAs typically reside in the nucleus, with their intranuclear localisation temporally regulated according to the cell cycle [5, 59]. Retention of ecDNAs in cancer cells ultimately relies on their ability to combat selection pressures; ecDNAs must provide a fitness advantage to persist [21]. If this is not the case, ecDNAs are lost over time, as exemplified for *Dhfr +* ecDNAs cultured in the absence of methotrexate [29, 31]. It therefore appears that ecDNA survival largely depends on transcriptional output. The open chromatin structure of ecDNAs [8] allows transcriptional machinery to more readily access resident genes [9], with transcriptional efficiency also increased through gene enhancer rewiring/hijacking. More specifically, the arbitrary manner in which chromosome fragments ligate to form ecDNAs profoundly reroutes gene regulatory circuits. This unique property afforded by DNA circularisation may result in the juxtaposition of oncogenes alongside chromosomally distant gene enhancers [65]. Co‐amplification of enhancers with oncogenes further drives oncogene expression [66], with some studies suggesting that enhancers not only increase ecDNA transcription, but may be required to exert this function [65]. Consistent with this, positive selection of glioblastoma cells harbouring ecDNAs not only depends on extrachromosomal *EGFR* amplification, but also on the co‐amplification of two upstream enhancers [65]. Such complex rewiring of gene regulatory circuits is also observed in neuroblastoma, where Helmsauer *et al*. [43] reported that the distally located enhancer, e4, is proximal to *MYCN* in ~90% of ecDNA species. Transcriptional efficiency of ecDNAs can therefore be enhanced through various means to provide cells with a selective advantage that results in ecDNA retention.

Just like chromosomal DNA, ecDNAs replicate once per cell cycle, solely within S phase [24]. EcDNA replication occurs autonomously, and while it appears that chromosomal DNA replication machinery is involved, ecDNA‐specific mechanisms may also contribute [9]. This idea is supported by the observation that ecDNAs dissociate from chromosomes during replication and migrate from the periphery towards the centre of the nucleus [5, 67]. The dynamics of ecDNA replication are also distinct: In contrast to the well‐synchronised replication of chromosomal DNA, ecDNAs replicate at various stages throughout S phase, with replication fork velocity markedly slower [68]. By implementing CRISPR‐C to generate *EGFR*‐harbouring ecDNAs in human glioblastoma (U251) and epithelial kidney (HEK‐293T) cell lines, Kang *et al*. [69] identified several factors that orchestrate ecDNA replication. Genes involved in chromosomal DNA replication, such as *PCNA*, *MCM2*, *POLD*, and *RPA1*, were all shown to be significantly enriched in these cell lines. Activation of various DNA repair pathways is also linked to ecDNA replication and maintenance. Topoisomerases relieve DNA entanglements, such as supercoils and catenanes [70], which occur during replication through formation of reversible topoisomerase cleavage complexes (TOPCCs). However, TOPCCs occasionally become abortive, inducing DSBs within replicating DNA [70]. Consistent with a role for topoisomerases in ecDNA replication, *TOP2B* levels were found to be significantly enriched in ecDNA‐harbouring cell lines. Likewise, abortive TOP1 cleavage complexes (TOP1CC) were shown to colocalise to ecDNA, implying a role for TOP1 in ecDNA replication. Components of the alternative non‐homologous end joining (aNHEJ) DNA repair pathway rescue ecDNA from TOPCC‐induced damage, with LIG3, MRE11, and POLθ promoting ecDNA maintenance through the repair of replication‐induced DSBs. The ATM‐mediated DNA damage response (DDR) pathway also appears critical to ecDNA survival, with inhibition of this pathway leading to gross ecDNA depletion within cancer cells [69].

**Fig. 2:**
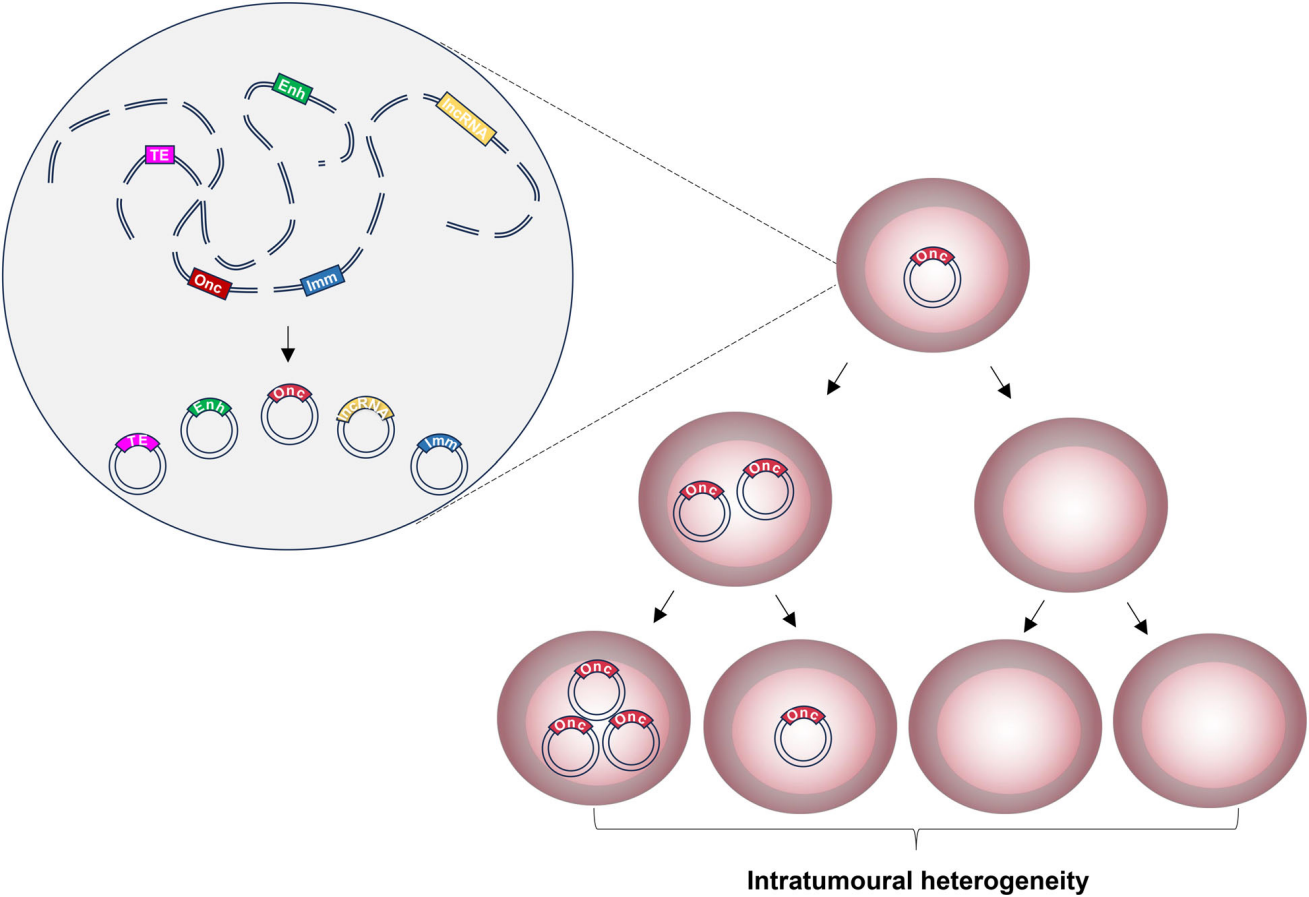
Schematic depicting the formation and inheritance of ecDNAs during cell division. EcDNAs may form through chromothripsis and can harbour oncogenes and/or other coding/non‐coding elements. For simplicity, single element‐containing species are shown, but complex forms harbouring both oncogenes and regulatory elements typically exist. Following generation, ecDNAs replicate once per cell cycle and are unevenly segregated to daughter cells at mitosis. This results in significant tumour heterogeneity through time. Abbreviations: Enh, enhancer; Imm, immunomodulatory gene; lncRNA, long non‐coding RNA; Onc, oncogene; TE, transposable element. Figure partially created in BioRender. Wilson, E. (2025) https://BioRender.com/7x9g3sm.

Taken together, these studies suggest that while ecDNAs may indeed hijack chromosomal DNA replication machinery, the process involved is distinct. Furthermore, while chromosomal DNA is evenly inherited at mitosis, ecDNA segregation is often unequal [19]. This means that while some daughter cells receive none, others receive multiple ecDNAs (Fig. 2). Recent work has shown that regulatory elements encoded by ecDNAs help mediate their effective segregation during mitosis. Depletion of the long non‐coding RNA (lncRNA), *PVT1*, which is frequently co‐amplified on *MYC*‐harbouring ecDNAs, precludes chromosomal hitchhiking in COLO320‐DM and PC3 cell lines [71]. This suggests that ecDNAs are not mere passengers of the segregation process but rather play an active role in ensuring their faithful inheritance during cell division. Uneven ecDNA segregation drives intratumoural heterogeneity and leads to the emergence of high copy‐number clones that accelerate cancer progression [72]. Increased oncogene expression leads to clonal expansion, resulting in a highly evolved tumour with an abnormally high ecDNA burden [19, 38, 46]. Conversely, if selection pressures render ecDNAs detrimental, as in the case of *EGFR*‐harbouring ecDNAs in the context of *EGFR*‐targeting therapy [73, 74], cells which have sequestered their ecDNAs in the form of HSRs become the predominant clones [21]. Moreover, while the case for a singular ecDNA species is described above, multiple species frequently coexist within cancer cells, adding further complexity to segregation dynamics and tumour evolution [75]. This complexity is reinforced by the evolution of ecDNA species themselves during cancer progression [74]. More specifically, coexisting ecDNA species have been proposed to morph together within cells, as has been shown for *KRAS*‐harbouring ecDNAs in OAC [8, 21, 63]. EcDNA evolution is also evident between diagnosis and relapse [74], with the acquisition of secondary somatic mutations within ecDNA coding sequences found to potentiate therapy resistance at relapse in ecDNA‐harbouring glioblastomas [76]. Taken together, these data demonstrate that ecDNAs are not static components within cancer cells but instead show a high degree of plasticity that allows them to readily evolve in line with disease progression.

## Contemporary advances in ecDNA research

Novel mechanisms through which ecDNAs promote tumour progression are continually being uncovered. EcDNAs move freely within nuclei, and along with the presence of enhancer cargo, have led to the suggestion that they also act as mobile transcriptional enhancers [77]. Evidence for this was provided by Zhu *et al*. [78], who employed chromatin interaction analysis with paired‐end‐tag (ChIA‐PET) sequencing to interrogate the ecDNA‐chromatin interactome. By capitalising on the association between ecDNAs and RNA polymerase II (RNAPII), a vast network of ecDNA‐chromatin interactions was identified. Contact sites between ecDNAs and chromosomes are preferentially located at enhancers and promoters, respectively. In addition, many ecDNA‐resident enhancers are super‐enhancers [75], with known oncogenes being significantly enriched among chromosomal gene targets. Together, these findings indicate that ecDNAs may have roles as *trans‐*regulatory elements, increasing the expression of chromosomal oncogenes [79].

Recent advances in imaging techniques have revealed that ecDNAs cluster within cells to form ecDNA hubs [72, 80]. These hubs typically comprise 10–100 ecDNAs and have been reported in several cancer types. By focussing on *MYC*‐amplified ecDNAs, Hung *et al*. identified a correlation between ecDNA clustering and increased *MYC* transcription, even after normalising for copy number. This indicates that ecDNAs within hubs are more actively transcribed than their isolated counterparts [80]. Additionally, hub‐residing ecDNAs appear to interact with each other, raising the intriguing possibility that enhancers on one ecDNA can stimulate oncogene expression on another. Indeed, this paradigm is further supported by the presence of enhancer‐only ecDNAs within some cancers [42]. However, ecDNA hub formation requires further exploration, as super‐resolution imaging of ecDNA within primary glioblastoma cells failed to detect this phenomenon [81]. One possible explanation for such disparities lies within size differences of the ecDNAs analysed, with those contained within primary glioblastoma cells [81] notably smaller than those predisposed to hub formation in other cancers [80].

While a link with disease progression is well‐established, recent evidence suggests that ecDNAs arise before overt cancer development. BO is a precancerous condition which progresses to OAC in 3–5% of cases [82]. Luebeck *et al*. [44] examined WGS data from 266 BO patients and found a strong correlation between ecDNA levels and the stage of BO‐OAC progression. Whereas no ecDNAs were identified in patients with nondysplastic BO/low‐grade dysplasia, ecDNAs were identified in 18% of patients with high‐grade dysplasia. This figure rose to 25% in patients with early‐stage OAC and to 43% in patients with late‐stage OAC [44]. These findings demonstrate that ecDNAs may hold predictive power in some malignancies, potentially allowing patient stratification before cancer develops.

Although extensive progress has been made concerning ecDNA contribution to cancer progression, the story is by no means complete. Given the well‐established link between ecDNAs and poor patient outcomes, development of ecDNA‐targeting therapies is crucial. This represents an ongoing challenge, with efforts focussed mainly on exploiting mechanisms integral to ecDNA survival [5]. The increased transcription and replication occurring in ecDNA‐harbouring cells induces transcription‐replication conflict. Collision of RNAPII with DNA replication machinery leads to replication fork stalling that is relieved through the action of checkpoint kinase 1 (CHK1). Inhibition of CHK1 in ecDNA‐harbouring cells induces cell death, exposing a synthetic lethality that holds promising therapeutic potential [83]. Disruption of ecDNA hubs is also being explored. The bromodomain and extraterminal domain (BET) protein, BRD4, acts as a molecular adhesive that maintains hub stability, while also mediating effective ecDNA segregation at mitosis [71]. Use of the BET inhibitor, JQ1, effectively disperses ecDNA hubs [80], offering another approach through which ecDNAs may be targeted. The recently uncovered dependency of ecDNAs on DDR factors may represent a third therapeutic avenue, with ecDNA‐harbouring cells displaying heightened sensitivity to both ATM and CHK2 inhibition [69]. Clearly, the identification of an ecDNA‐targeting therapy would reshape the current cancer landscape and provide an invaluable weapon to combat these molecules in clinical settings.

## Excised signal circles (ESCs): novel cancer‐associated eccDNAs

In contrast to ecDNAs, ESCs were long believed to be inert forms of eccDNA. However, growing evidence suggests that ESCs are also cancer‐associated and share more similarities with ecDNAs than previously thought. These similarities are not confined to structure and physical shape but also relate to function, with both molecules contributing to the development and progression of human disease [8, 13, 16, 18, 44, 45].

## The formation of ESCs: V(D)J recombination

**Fig. 3:**
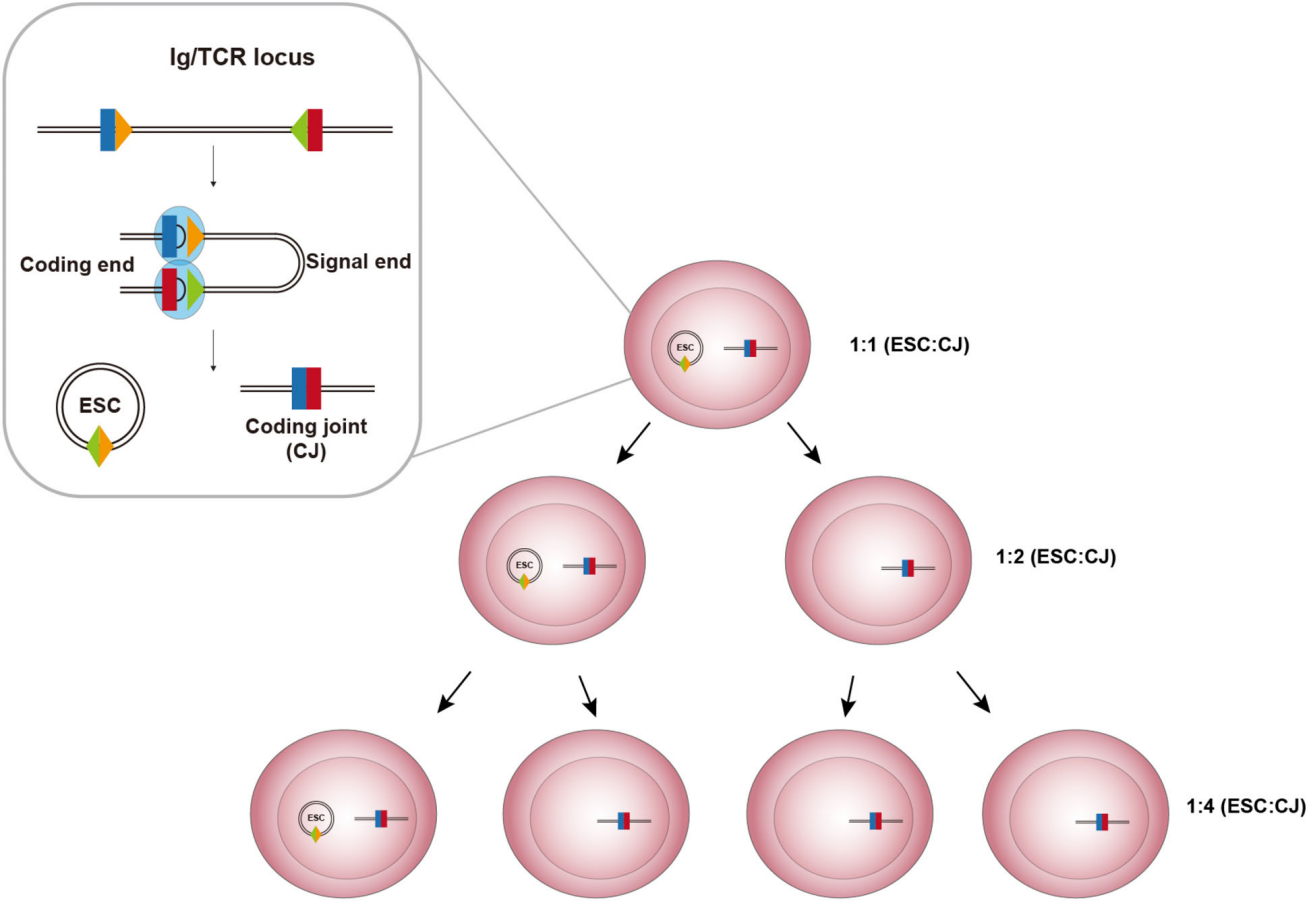
ESC formation through V(D)J recombination at the immunoglobulin (*Ig*) and T‐cell receptor (*TCR*) loci. After recognising RSSs (yellow and green triangles) proximal to gene segments (blue and red rectangles), RAG proteins (blue ovals) generate DSBs at the gene segment‐RSS heptamer junction. The four resulting broken ends are subsequently shepherded to the cNHEJ DNA repair pathway [86]. Joining of the coding ends forms a coding joint (CJ), whereas DNA repair of the signal ends forms a signal joint (SJ) that is expelled as an excised signal circle (ESC). Under normal conditions, the CJ is stably present in the genome, whereas the ESC was long thought to be invariably diluted at mitosis. The ESC:CJ ratio therefore begins at 1 : 1 and was predicted to be halved with each cell division. Figure partially created in BioRender. Wilson, E. (2025) https://BioRender.com/rqsroy7.

While several models have been proposed to explain ecDNA formation, only one mechanism generates ESCs: V(D)J recombination (Fig. 3). This reaction is strictly confined to developing lymphocytes and generates an extensive repertoire of antigen receptors in B and T cells [84], allowing the adaptive immune system to combat the millions of pathogens that may be encountered throughout a lifetime. Such vast antigen receptor diversity is generated by the somatic recombination of individual gene segments within the immunoglobulin (*Ig*) and T‐cell receptor (*TCR*) loci (V, D, and J gene segments at the *IGH* and *TRB*/*TRD* loci, and V and J gene segments at the *IGK*/*IGL* and *TRA*/*TRG* loci) [85]. Although the selection of individual gene segments is largely stochastic, the reaction itself is precisely targeted, resulting in recombination only at specific regions of the genome [85].

The specificity of V(D)J recombination is governed by the recombination‐activating gene 1 (RAG1) and 2 (RAG2) proteins, which form a heterotetrameric recombinase complex that recognises and cleaves DNA at recombination signal sequences (RSSs) [86, 87]. RSSs lie proximal to each gene segment and comprise conserved heptamer (5′‐CACAGTG) and nonamer (5′‐ACAAAAACC) sequences separated by a nonconserved spacer of either 12 (12‐RSS) or 23 bp (23‐RSS). The recombinase first binds to a 12‐ or 23‐RSS to form a signal complex (SC), before capturing a partner RSS of a different spacer length to form a paired complex (PC) [88]. Coupled RSS cleavage is then initiated by RAG1 triggering single‐strand nicks at each coding segment‐heptamer junction. The resulting 3′‐hydroxyl (3′‐OH) groups attack their opposite DNA strand in direct transesterification reactions, generating two DSBs, each with one blunt signal end and one closed hairpin coding end. These remain bound by the recombinase complex, forming a postcleavage complex (PCC) [86] that shepherds the four broken DNA ends to the classical nonhomologous end joining (cNHEJ) DNA repair pathway.

The final step in V(D)J recombination involves joining of the DNA ends: While the blunt signal ends are joined directly to form a signal joint (SJ), the coding ends undergo extensive processing before joining. This involves hairpin opening, followed by addition or deletion of bases prior to formation of the final coding joint (CJ) [85]. While the CJ remains integral to the genome and encodes the variable region of the antigen receptor, the SJ is usually expelled as an ESC [89]. ESCs contain the 12‐ and 23‐RSSs in a head‐to‐head configuration (i.e. the SJ), as well as the DNA from in between the newly joined gene segments, which can vary from 650 bp to 1 Mb. Indeed, depending on the locus, or locus region, from which they arise, these molecules are referred to as TCR‐rearrangement excision circles (TRECs), BCR‐rearrangement excision circles (BRECs) or kappa‐deleting recombination excision circles (KRECs) [11, 13, 90]. The term ESC encompasses all such circular DNAs generated through antigen receptor rearrangement, and owing to a lack of functional genes, these molecules were long thought to be inert entities with no specific function. Indeed, the joining of DNA ends to form the ESC was suggested to serve a protective role by preventing potentially reactive signal ends from participating in aberrant reactions [91]. Despite this apparent benefit, however, it is now known that far from being inert, ESCs contribute to genome instability *in vivo* [13, 14, 15, 16, 18].

## ESCs are linked to genome instability

**Fig. 4:**
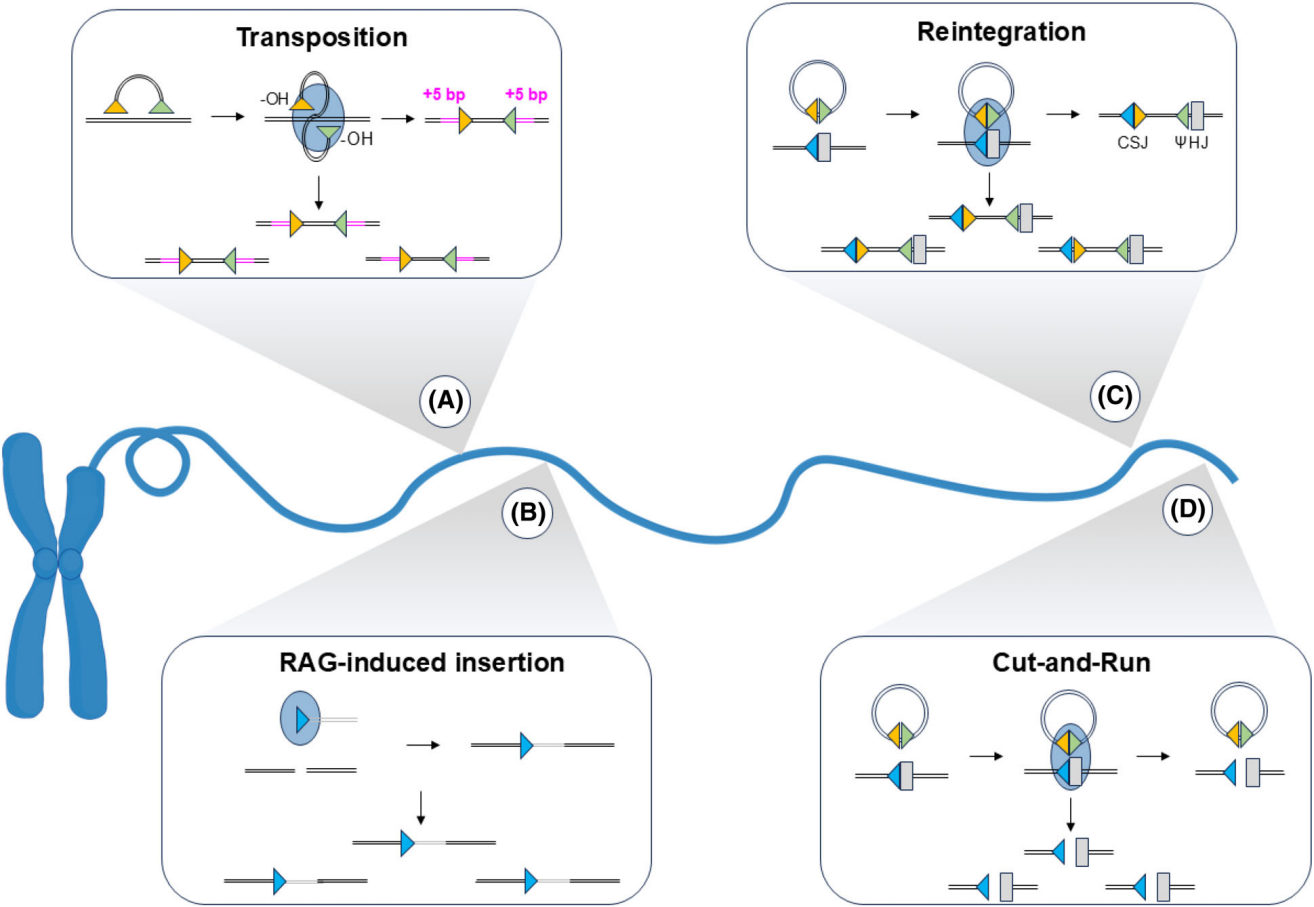
Four mechanisms through which V(D)J recombination by‐products cause genome instability. (A) Transposition: RAG proteins utilise the free hydroxyl (‐OH) groups on signal ends to nonspecifically attack opposing sides of DNA via a transesterification reaction. The signal ends are subsequently inserted into the DNA, with DNA repair generating 5 bp duplications flanking each end of the transposition site. (B) RAG‐induced insertion: RAG‐mediated cleavage products are inserted into RAG‐independent breaks throughout the genome. Inserted DNA may comprise signal ends with RSSs flanking the DNA, coding ends which lack RSSs, or hybrid ends (shown) with an RSS on one side of the DNA. (C) Reintegration: RAG proteins bind to an ESC SJ and form a PC with a genomic cRSS. Symmetric cleavage within the PC generates four broken ends which recombine to cause ESC reintegration. Sites of reintegration are marked by the presence of chromosomal signal joints (CSJs) and pseudo‐hybrid joints (ψHJs). (D) Cut‐and‐Run: RAG proteins bind to an ESC SJ and form a PC with a partner cRSS. Asymmetric cleavage results in DSB formation at the cRSS only. The intact ESC is then free to interact with additional cRSSs and trigger further DSBs. Rectangles represent coding segments, triangles represent RSSs/cRSSs, and blue ovals represent RAG proteins. Figure partially created in BioRender. Wilson, E. (2025) https://BioRender.com/6n3ja46

V(D)J recombination by‐products were first proposed to threaten genome stability via RAG‐mediated transposition (Fig. 4A) [92]. *In vitro* experiments demonstrated that RAG proteins insert cleaved signal ends into DNA in a sequence nonspecific manner. This reaction is akin to that of cut‐and‐paste transposons and generates a 5 bp duplication at either end of the transposition site [92, 93]. However, the biological significance of transposition appears negligible, with very few cases documented *in vivo* [94].

Although the dangers of transposition seem insignificant, V(D)J recombination by‐products mediate genomic damage in other ways. Using a mouse model, Rommel *et al*. [95] showed that DNA excised by RAG proteins can be inserted into DSBs in the *c‐myc* oncogene. This reaction differs from transposition in that although the excised fragment is generated by RAG cleavage, the sites of insertion are generated by RAG‐independent mechanisms (Fig. 4B). Moreover, signal ends are not the sole substrates for this reaction, with excised coding ends that lack RSSs, as well as hybrid ends with a single RSS, also found to be inserted at RAG‐independent breaks. Indeed, WGS analysis has revealed that such RAG‐induced insertions occur in human cancer, with ~15% of the follicular lymphoma (FL) and acute lymphoblastic leukaemia (ALL) patients analysed harbouring *Ig*/*TCR* fragment insertions at various genomic loci [95].

While the risk posed by linear V(D)J recombination by‐products is partly mitigated by their circularisation to form ESCs, ESC SJs can be re‐bound and cleaved by RAG proteins, reigniting the threat to lymphocyte stability. One outcome of this is ESC reintegration [13], which typically occurs at RSSs and RSS‐like sequences, known as cryptic RSSs (cRSSs), that lie outside the antigen receptor loci (Fig. 4C). More than six million cRSSs are present within the human genome [96], and while not all are functional [97], these sequences are substrates for off‐target RAG cleavage, resulting in indels or chromosomal translocations [17, 98]. During reintegration, a RAG/ESC complex associates with a genomic RSS/cRSS, followed by coupled cleavage to open both the ESC and RSS/cRSS. This generates four broken ends, which are joined to reintegrate the ESC into the genome. The effects of this reaction largely depend on its location, with reintegration at proto‐oncogenes or tumour suppressor genes potentially leading to cell dysregulation. Vanura *et al*. [13] explored this using plasmid models harbouring cRSSs that flank the *LMO2* and *TAL2* proto‐oncogenes. Remarkably, the reintegration efficiency at cRSSs was comparable to that of an authentic RSS. Furthermore, a next‐generation sequencing (NGS) analysis has since identified TREC reintegration within genes associated with T‐ALL and T‐cell lymphoblastic lymphoma (T‐LBL). *ZFP36L2*, a tumour suppressor gene, was found to be a reintegration hotspot, with 1% of the T‐ALL/T‐LBL patients investigated harbouring TREC reintegration events within this gene [15]. Moreover, many patients with TREC reintegration lack T‐ALL/T‐LBL driver gene mutations, suggesting a role for reintegration in leukaemogenesis [15].

More recently, a novel mechanism through which ESCs threaten lymphocyte stability has been discovered [16]. Cut‐and‐run differs from the aforementioned reactions in that reinsertion of excised DNA does not occur, but instead, the RAG/ESC complex triggers DSBs at RSSs/cRSSs throughout the genome (Fig. 4D). This mechanism was first proposed based on *in vitro* cutting assays, which showed that in the presence of ESC SJs, RAG proteins efficiently cleave both 12‐ and 23‐RSS‐containing oligonucleotides, but the SJ is cleaved significantly less [16]. This asymmetric cleavage has stark implications for genome integrity, suggesting that ESCs may trigger breaks at one cRSS, relocate and trigger further breaks at another cRSS. Indeed, when co‐present with RAG proteins, RAG/SJ complexes generated a significant number of DSBs in human cell lines [16]. Subsequent analysis of these DSBs using linear amplification‐mediated high‐throughput genome‐wide translocation sequencing (LAM‐HTGTS) gave the surprising result that many map to *bona fide ETV6::RUNX1+* BCP‐ALL breakpoints [16, 17], and also colocalise to known cancer driver genes. Together, these results suggest a role for cut‐and‐run in BCP‐ALL development. Indeed, as is further discussed below, a strong link between cut‐and‐run and BCP‐ALL has since been uncovered [18], unequivocally demonstrating that ESCs are cancer‐associated eccDNAs.

## Elucidating the true impact of ESCs

Since their discovery in 1987 [99], ESCs have been characterised as non‐replicative entities that are invariably diluted through cell division [90]. In stark contrast to many ecDNAs, cells in which specific ESCs are generated can be traced by virtue of the corresponding CJ footprint that is retained within the genome. Taking these characteristics at face value, several studies have employed ESC:CJ ratios as a proxy to monitor lymphocyte naivety [90]. By assuming an initial 1:1 ratio that decreases exponentially through mitosis, Markert *et al*. [100] utilised TREC levels to monitor T‐cell output following thymus tissue transplant in patients with congenital athymia. Other scenarios in which ESC:CJ ratios have been utilised include measuring loss of thymic function in HIV‐1 infection [101], post‐transplant recovery in multiple myeloma (MM) [102], and newborn screening for primary immunodeficiency [103].

While such studies have been valuable, they assume that ESCs are non‐replicative and diluted at each cell division. In support of this idea, ESCs were found to be gradually lost in actively recombining T cells during mitosis [104]. Although contrasting data emerged in 1995 when Livak and Schatz showed that ESCs were sustained at high levels in peripheral mouse thymocytes [105], a replicative mechanism was not proposed due to their apparent exclusivity to nondividing cells. Indeed, this perceived inability to replicate was furthered by early reports suggesting a lack of ESC replication origins [105, 106]. However, core replication origins have since been mapped to regions of the *IGK* and *IGL* loci from which many ESCs derive [18]. Further, while TRECs, which are produced exclusively in the adult thymus, have a half‐life of only 2 weeks in chickens, TRECs in rhesus macaques have been detected a year post‐thymectomy [107]. In humans, ESCs have been detected up to 39 years after this same procedure [101]. Although such observations challenge the idea that ESCs are invariably lost through mitosis, the true replicative potential of ESCs has only recently been explored. Through in‐depth analysis of both normal mouse lymphocytes and human BCP‐ALL patient samples, Gao *et al*. demonstrated for the first time that ESCs can replicate. By analysing ESC:CJ ratios during progressive stages of mouse B‐cell development (pre‐B, bone marrow IgM^+^, spleen IgM^+^, and spleen IgG^+^), it was first shown that ESC:CJ ratios remain relatively stable, or even increase, between pre‐B and IgG^+^ lymphocyte populations [18]. Indeed, given that lymphocytes undergo at least six cell divisions during IgM^+^ to IgG^+^ maturation [108], the ESC:CJ ratio in IgG^+^ cells is expected to fall to < 2% of its starting level in the absence of ESC replication.

Prompted by the intriguing findings in mice, ESC replication was then investigated in BCP‐ALL, where ESCs have been linked to mutational events [16]. Using a combination of high‐throughput sequencing, PCR, and imaging approaches, ESCs were identified in almost all patients, though the levels varied considerably [18]. Definitive evidence of ESC replication was subsequently provided through incorporation of the thymidine analogue, 5‐bromo‐2′‐deoxyuridine (BrdU), in cells that had passed through only one S phase [109]. Just as for experiments that showed ecDNA replication [24], the presence of labelled ESCs in metaphase chromosome spreads also confirmed the replication of ESCs [18].

A key finding from the studies of Gao *et al*. [18] is the correlation between heightened ESC replication and BCP‐ALL relapse. Analysis of ESC levels in the diagnosis samples of BCP‐ALL patients who do and do not progress to relapse revealed highly significant increases in those who eventually relapse. Given that ESCs compromise genome integrity [13, 14, 16], patient sequences were subsequently analysed for structural variants (SVs) indicative of cut‐and‐run and reintegration. Data from 150 BCP‐ALL patients revealed that cut‐and‐run events underpin ~24% of all SVs and that these events occur significantly more often in patients who progress to relapse [18]. Further, like ecDNAs, the presence of ESCs is linked to clonal expansion, and in line with earlier reports in humans and other primates [101, 107], many ESCs were shown to persist for several years [18]. Together, these findings revert the long‐held dogma regarding their inert nature and collectively show for the first time that, like ecDNAs, ESCs truly contribute to cancer progression.

## Tying the knot: the newfound relationship between ecDNAs and ESCs

EcDNAs have long been thought of as the sole cancer‐associated eccDNA, facilitating increased tumour aggression and reduced patient survival. Over 60 years since their discovery, the precise ways in which these molecules promote cancer development are still being uncovered [46]. Nonetheless, extensive research has generated a vivid blueprint of their oncogenic role. Central to this is gross copy‐number amplification of oncogenes and regulatory elements in extrachromosomal form. Multiple ecDNA species can coexist within single cells, harbouring oncogenes, immunomodulatory genes and various regulatory elements [39, 40, 41, 42, 43]. The juxtaposition of ectopic enhancers proximal to oncogenes facilitates increased transcription, which is further bolstered by the increased chromatin accessibility of circular DNA [43, 110]. Crosstalk between ecDNA species within hubs may further promote oncogene expression, laying the groundwork for highly aggressive cancers that increase the risk of mortality [80, 111].

Discovering the precise role of ESCs in cancer development has been much less straightforward. Long assumed to be innocuous by‐products of V(D)J recombination, the dangers that these molecules pose have only recently come to light. Reintegration and cut‐and‐run threaten lymphocyte stability [13, 14, 15, 16], but the apparent non‐replicative nature of ESCs seemingly mitigated the risk posed *in vivo*. It is now known that ESCs replicate [18], and much like ecDNAs, contribute to cancer progression. Recent work has bridged the gap between mechanistic studies and true disease‐causing potential, with ESCs now intrinsically linked to the relapse of BCP‐ALL [18]. With these discoveries, the relationship between ecDNAs and ESCs can finally be appreciated.

**Fig. 5:**
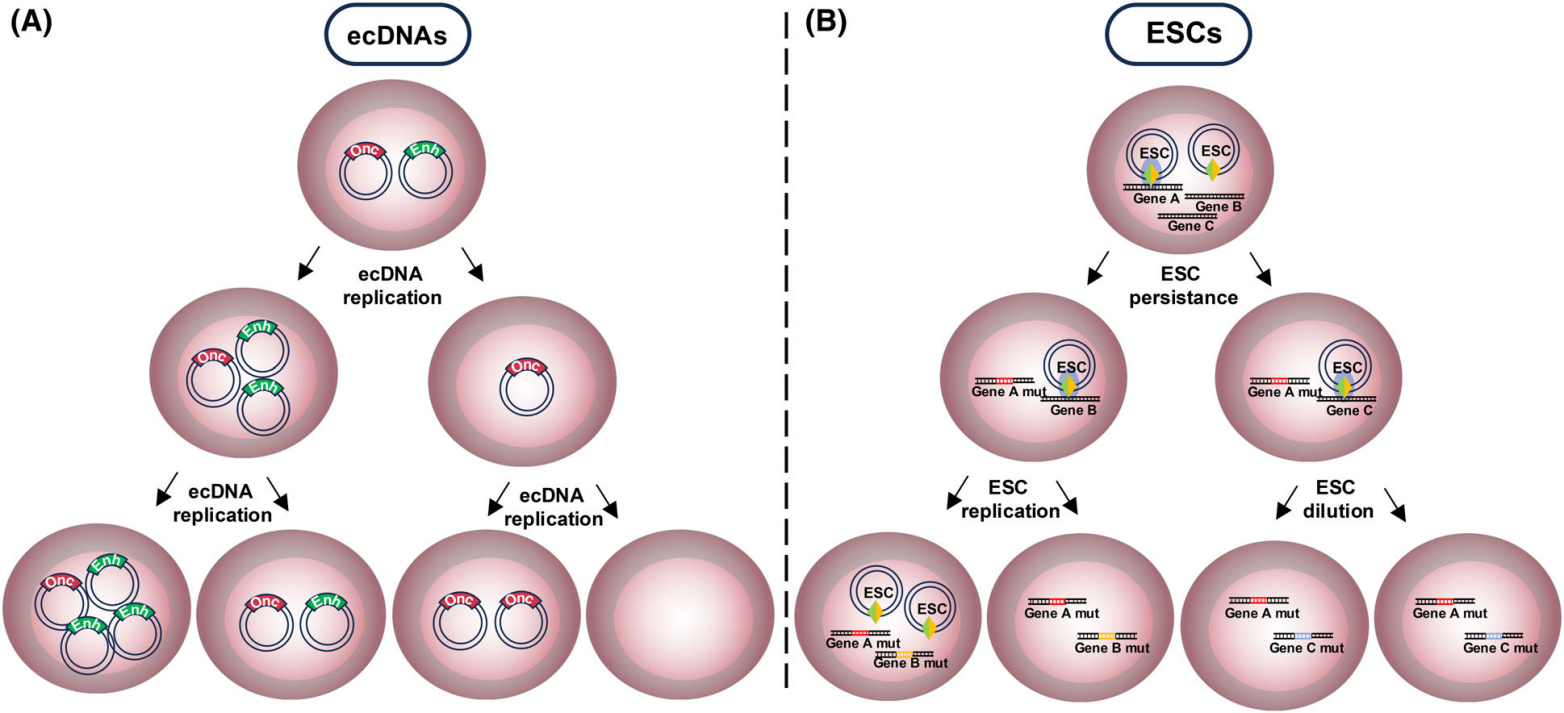
Intracellular behaviour and inheritance patterns of ecDNAs and ESCs in cancer. (A) Multiple ecDNA species may coexist within single cells, harbouring oncogenes (Onc) and/or regulatory elements such as enhancers (Enh). EcDNAs replicate once per cell cycle, with unequal segregation at mitosis leading to intratumoural heterogeneity. EcDNA presence is required for disease progression, meaning that ecDNAs must continually replicate to confer a growth advantage to cancer cells. (B) Multiple ESCs may coexist within cells where they form a complex with RAG proteins to trigger mutations at cancer driver and relapse‐associated genes. The targeting of various genes throughout the genome leads to the emergence of subclones with differing mutations. Mutated genes (mut) are inherited by all daughter cells and remain irrespective of whether ESCs persist, replicate, or are diluted at mitosis. Figure partially created in BioRender. Wilson, E. (2025) https://BioRender.com/bxmj2x2.

Structurally, both molecules exist as extrachromosomal circles and are among the largest forms of eccDNA within human cells [112]. Replicative in nature, both molecules trigger clonal expansion when present at elevated levels [7, 18, 38]. Although this leads to worse disease outcomes in both instances, the molecular basis is largely dissimilar (Fig. 5A,B). EcDNAs frequently promote cancer through oncogene amplification [45], whereas ESCs participate in catalytic reactions that cause gene mutations [18]. Unlike ecDNAs, ESCs rely on the copresence of effector molecules—the RAG proteins—to exert damaging effects. This explains why ESCs themselves are compatible with healthy cells, whereas ecDNAs are predominantly associated with cancer [1, 113]. Such mechanistic differences are also reflected in cellular distribution. Light microscopy and DNA FISH have been used to study ecDNA distribution [114], with several studies demonstrating their gross accumulation, often > 100 copies [44, 68, 114], in cancer cells. DNA FISH studies of ESCs in BCP‐ALL have also demonstrated that ESCs accumulate in single cells [18]. However, ESC levels are far lower at 1–10% of that of ecDNAs, presumably because their presence is not required to sustain a growth advantage and the mutational burden remains even if ESCs are lost.

Although ecDNAs are linked to the progression of several cancers, the lymphocyte‐restricted nature of V(D)J recombination means that ESCs are confined to haematological malignancies of lymphoid origin. Interestingly, these diseases are among the few forms of cancer where ecDNAs are not particularly well‐documented. Although studies have identified ecDNAs in AML, diffuse large B‐cell lymphoma (DLBCL), mantle cell lymphoma (MCL) and APML [62, 115], they are not readily detectable in acute lymphoblastic leukaemias such as BCP‐ALL and T‐ALL. While the reasons for this are not clear, ESCs appear to fill this void by assuming the role of a circular DNA that promotes disease progression. Indeed, much research on the contribution of ESCs to leukaemia development has centred on those arising from the *IGK*/*IGL* and *TCR* loci (Table 2) [15, 18]. However, ESCs are also formed through V(D)J recombination at the *IGH* locus in B cells, with other circular DNAs generated at this locus via class‐switch recombination (CSR) [116]. Given the recently identified role of *IGK*/*IGL* ESCs in BCP‐ALL, investigation into ESCs derived from other recombination events is needed to delineate the entire spectrum of ESC‐associated disease. Further, the roles of circularised nucleic acids in ALL development extend beyond the realms of DNA, with circular RNAs (circRNA) also being implicated [117]. CircRNAs are single‐stranded, covalently closed RNAs, which form through back‐splicing events within messenger RNA (mRNA). Ubiquitous in nature, circRNAs mediate genomic instability through R‐loop formation whereby their binding to cognate DNA displaces a loop of single‐stranded DNA (ssDNA) [117, 118]. Studies in BCP‐ALL have shown that R‐loops are commonplace within the *KMT2A* (*MLL*) gene, and may even instigate the formation of *KMT2A* translocations in *KMT2A‐*rearranged (*KMT2A‐*r) BCP‐ALL [117].

**Table 2:** ESCs and circularised DNAs/RNAs found in lymphocytes and the mechanisms by which they contribute to lymphoproliferative malignancies.

Lineage	Circular DNA/RNA	Formation	Malignancy	Mechanism	References
B cells	*IGK* ESCs	By‐products of V(D)J recombination at the *IGK* locus	BCP‐ALL	Cut‐and‐run at tumour driver‐ and relapse‐associated genes	[16, 18, 91]
	*IGL* ESCs	By‐products of V(D)J recombination at the *IGL* locus	BCP‐ALL	Cut‐and‐run at tumour driver‐ and relapse‐associated genes	[16, 18, 91]
	*IGH* ESCs	By‐products of V(D)J recombination at the *IGH* locus	–	–	–
	CSR‐derived circular DNAs	By‐products of CSR at the *IGH* locus	–	–	–
	CircRNAs	Back‐splicing of mRNA	BCP‐ALL (*KMT2A‐r*)	Generation of chromosomal translocations through R‐loop formation	[117]
T cells	TCR ESCs	By‐products of V(D)J recombination at the *TCR* loci	T‐ALL/T‐LBL	Reintegration at proto‐oncogenes/tumour suppressor genes	[13, 15]

**Fig. 6:**
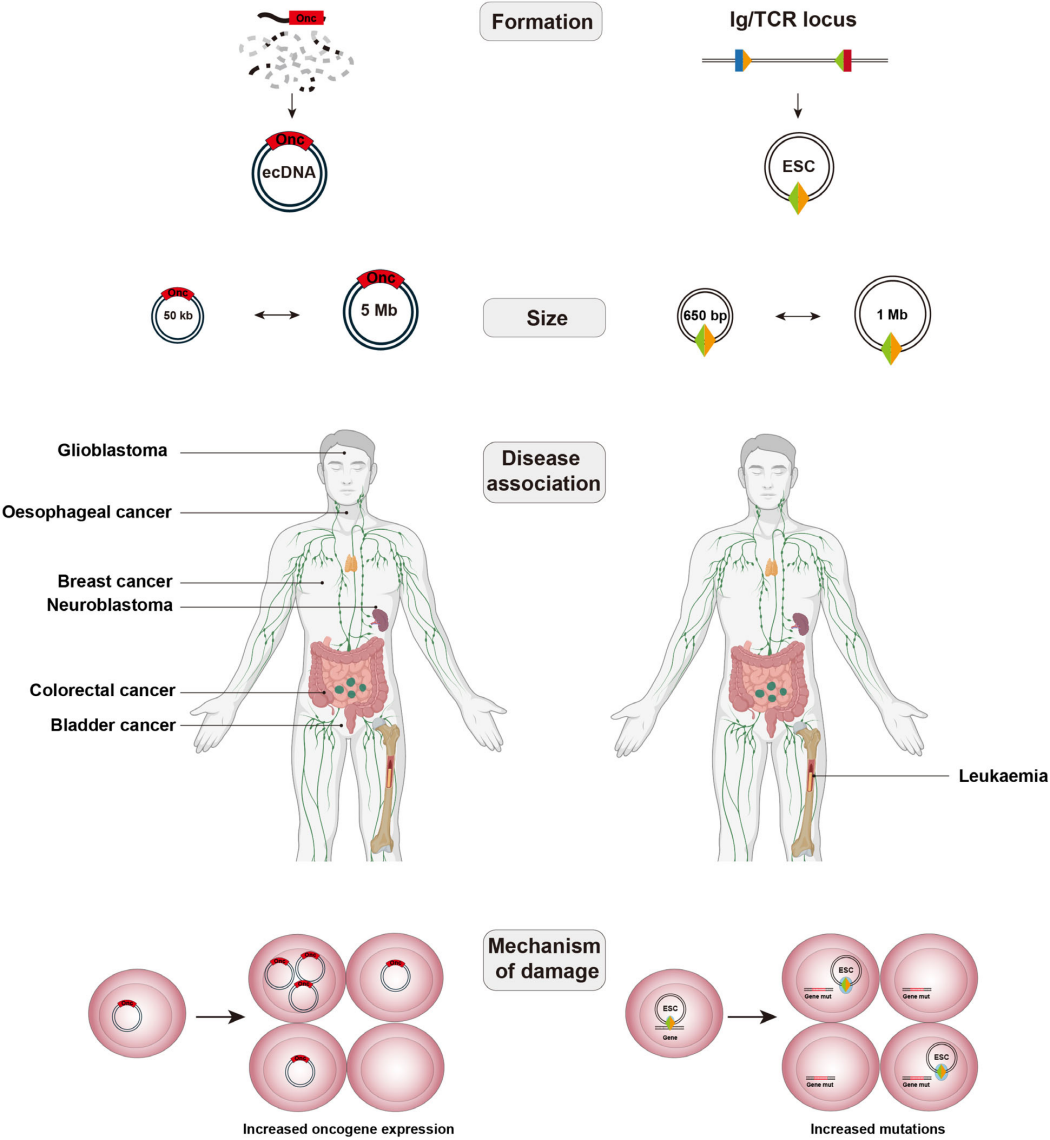
Comparison of the key differences between ecDNAs and ESCs. EcDNAs (left) often form via chromothripsis whereas ESCs (right) form via V(D)J recombination at the antigen receptor loci. Both molecules are large forms of eccDNA, with ecDNAs ranging between 50 kb and 5 Mb and ESCs between 650 bp and 1 Mb. EcDNAs have been observed in many cancers, whereas ESCs have only been associated with leukaemia. Although both molecules facilitate clonal expansion, ecDNAs typically impart this through oncogene amplification, whereas ESCs cause mutations throughout the genome. Figure partially created in BioRender. Wilson, E. (2025) https://BioRender.com/y1k75zg.

While there are several differences between ESCs and ecDNAs (Fig. 6), both molecules share many fundamental properties. Their similarly large size and circular conformation may mean that both molecules utilise related mechanisms for maintenance and replication. The factors involved in ecDNA replication are only now being revealed, with a clear dependency on chromosomal DNA replication and repair pathways [68, 69]. While even less is known about ESC replication, RNA‐seq data have identified several factors that may be involved. The homotrimeric PCNA protein acts as a sliding clamp that encircles replicating DNA [119]. *PCNA* is overexpressed in BCP‐ALL patients with elevated ESC levels [18], as well as in neuroblastoma cells harbouring ecDNAs, indicating a possible shared mechanism through which both molecules replicate [112]. The segregation dynamics of ESCs and ecDNAs may also bear resemblance, as indicated by imaging approaches showing marked intratumoural heterogeneity within cancer cells. Indeed, if further evidence confirms shared replication and/or segregation pathways, this could substantiate a combined effort to pharmacologically target both molecules. Pharmacological targeting of circular DNAs represents an exciting field, and efforts are well underway with regard to ecDNAs. The POTENTIATE trial is one such example, and is seeking to determine the effectiveness of the oral CHK1 inhibitor, BBI‐355, in treating ecDNA‐harbouring tumours [9, 120]. As mentioned earlier, CHK1 maintains cell integrity in spite of ecDNA‐induced replication stress, and the efficacy of the BBI‐355 inhibitor is being assessed both as a sole therapeutic agent as well as in combination with other targeted therapies such as erlotinib (EGFR inhibitor), futibatinib (FGFR1‐4 inhibitor) and BBI‐825 (ribonucleotide reductase inhibitor) [120, 121]. Preliminary data from this study have been encouraging, with preclinical models showing that BBI‐355‐erlotinib/BBI‐355‐futibatinib combinations exhibit potent antitumour activity [122]. If similar findings are recapitulated in patients, determining the extent to which proteins such as CHK1 are required for ESC maintenance could extend the therapeutic application of BBI‐355 to BCP‐ALL.

As well as exploiting synthetic lethalities imposed on cells through their presence, researchers are also exploring the possibility of targeting circular DNAs themselves using CRISPR‐based platforms. In cervical cancer, hybrid viral‐human ecDNAs are found, whereby the causative viral agent, human papillomavirus (HPV), is frequently identified within ecDNA sequences [9, 123, 124, 125]. This phenomenon has also been observed in HPV‐mediated oropharyngeal cancer (HPVOPCs), and originates from chromosomal integration of HPV DNA with subsequent excision and circularisation to form hybrid viral‐human ecDNA [126, 127]. By repositioning human enhancers proximal to viral sequences, the resulting hybrid ecDNAs are associated with increased expression of the viral oncoproteins E6 and E7. To counteract this, Nakagawa *et al*. [126] designed single‐guide RNAs (sgRNAs) that target the enhancer elements in hybrid ecDNAs and found that CRISPR interference (CRISPRi) technology indeed reduces expression of E6 and E7 in HPVOPC models. Similarly, by targeting unique breakpoint sequences within ecDNAs in other cancers, Pham *et al*. [128] have harnessed the type I‐E CRISPR‐Cas3 system to degrade ecDNA within cancer cells. Application of this system in COLO‐320 and GBM39 cells showed a reduction in ecDNA levels and tumour cell growth, highlighting its potential as a novel therapeutic approach [128]. Although the authors report a high degree of specificity, future studies will be necessary to minimise the dangers of off‐target effects associated with CRISPR‐based therapies. However, given that unique breakpoints are a shared feature among circular DNAs, the outlook remains promising, and such systems may eventually be repurposed to treat ESC‐associated diseases such as BCP‐ALL.

Finally, as both ecDNAs and ESCs are linked to disease progression when present at elevated levels, it is plausible that these molecules may serve as useful prognostic markers. The aforementioned studies in BO, where ecDNA presence is associated with progression to OAC [44], underscore a use for ecDNAs as cancer biomarkers. Similarly, for ESCs in BCP‐ALL, their presence at diagnosis appears strongly predictive of subsequent relapse, indicating a potential avenue for biomarker development [18]. Research into the identification of cancer biomarkers has soared in recent years, with the ultimate goal of being able to predict disease presence/outcome with minimal invasiveness and technical restraints [129]. Many efforts have focused on capitalising on the presence of cell‐free and circulating tumour DNA (cfDNA and ctDNA, respectively) in liquid biopsies of cancer patients. In small cell lung cancer (SCLC), where ecDNAs are present in ~20% of cases, researchers have shown that *MYC*‐amplified cell‐free ecDNAs are present in patient plasma [130]. Similar analyses in lung adenocarcinoma (LAC) patients have identified cell‐free circular DNAs within plasma samples, and although these were found to mainly comprise smaller eccDNAs (< 1 kb) [131, 132], such findings hold considerable promise given the highly invasive nature of current diagnostic tools [133]. The implementation of ESCs in a similar light seems feasible but requires further investigation given that their role in BCP‐ALL is linked to their presence in bone marrow aspirates. As such, determining whether high‐copy ESCs are present in peripheral blood samples of BCP‐ALL patients, either within malignant lymphoblasts or as cfDNA, is necessary. Indeed, owing to their intrinsic association with haematological malignancies, as well as their presence at low levels in the peripheral blood of healthy samples [18], it may be the case that ESCs are naturally a better fit than ecDNAs as liquid biopsy‐based biomarkers of cancer progression.

## Conclusion

In summary, this appears to be an important juncture in eccDNA research as we now know of two distinct forms that directly influence cancer progression. Although both ecDNAs and ESCs cause adverse disease outcomes, recent findings suggest that cancer‐associated eccDNA imparts an Achilles' heel on malignant cells. As such, future approaches exploiting such weaknesses may herald the birth of a new era in cancer treatment. However, as our understanding of ecDNA biology reigns vastly supreme to that of ESCs, this disparity first needs to be addressed to prevent leukaemia patients from lagging behind those with ecDNA‐associated malignancy. In an ideal scenario, future strategies targeting one cancer‐associated eccDNA could be leveraged to target another, simplifying the process of drug discovery and adding a potent tool to our therapeutic toolbox. For now, however, it is hoped that by at least appreciating the newfound relationship between ecDNAs and ESCs, we may soon see the tide begin to turn in the ongoing battle against circular DNA in cancer.

## Author contributions

DC wrote the manuscript with contributions from ZG and JB. The figures were prepared by ZG with contributions from DC and JB.
